# Case Report: Successful treatment with methotrexate in a 10-year-old boy with co-occurrence of generalized psoriasis and vitiligo

**DOI:** 10.3389/fimmu.2023.1255909

**Published:** 2023-10-03

**Authors:** Pui-Ying Leong, Tsu-Man Chiu, James Cheng-Chung Wei, An-Ping Huo

**Affiliations:** ^1^ Institute of Medicine, Chung Shan Medical University, Taichung, Taiwan; ^2^ School of Medicine, Chung Shan Medical University, Taichung, Taiwan; ^3^ Division of Allergy, Immunology and Rheumatology, Department of Internal Medicine, Chung Shan Medical University Hospital, Taichung, Taiwan; ^4^ Department of Dermatology, Chung Shan Medical University Hospital, Taichung, Taiwan

**Keywords:** psoriasis, vitiligo, methotrexate, JAK, biologics

## Abstract

The co-occurrence of psoriasis (PsO) and vitiligo is rare in Asian countries, especially in children. This case report presents the first-ever occurrence of PsO combined with vitiligo in an Asian boy under 6 years of age, in whom symptom improvement was observed after the use of methotrexate (MTX) as the sole treatment. Although previous studies have indicated that there is a close correlation between the two diseases, methotrexate (MTX), which is a commonly used treatment for PsO, is not a standard treatment for vitiligo. Even with advanced progress in biologics and Janus kinase inhibitor (JAKi), the biologics and JAKi used in vitiligo are still inconsistent. In our case report, the successful use of MTX indicated that there are shared immune pathways between PsO and vitiligo. Further exploration is needed to optimize the treatment options for this co-occurrence of PsO and vitiligo.

## Introduction

Psoriasis (PsO) is more prevalent in Northern European nations than in East Asia (1), and vitiligo is less common among Asian Americans than individuals of other races (2). Consequently, the co-occurrence of PsO and vitiligo is frequent in Europe and the Americas but rare in Asia, especially among children under 10 years of age. We present the first-ever case of an Asian boy under 6 years of age who developed PsO combined with vitiligo. This child’s symptoms improved after only methotrexate (MTX) was administered. Based on this treatment experience and relevant literature, we discuss the correlation between PsO and vitiligo and the therapeutic role of MTX in treating these two conditions.

**Figure 1:**
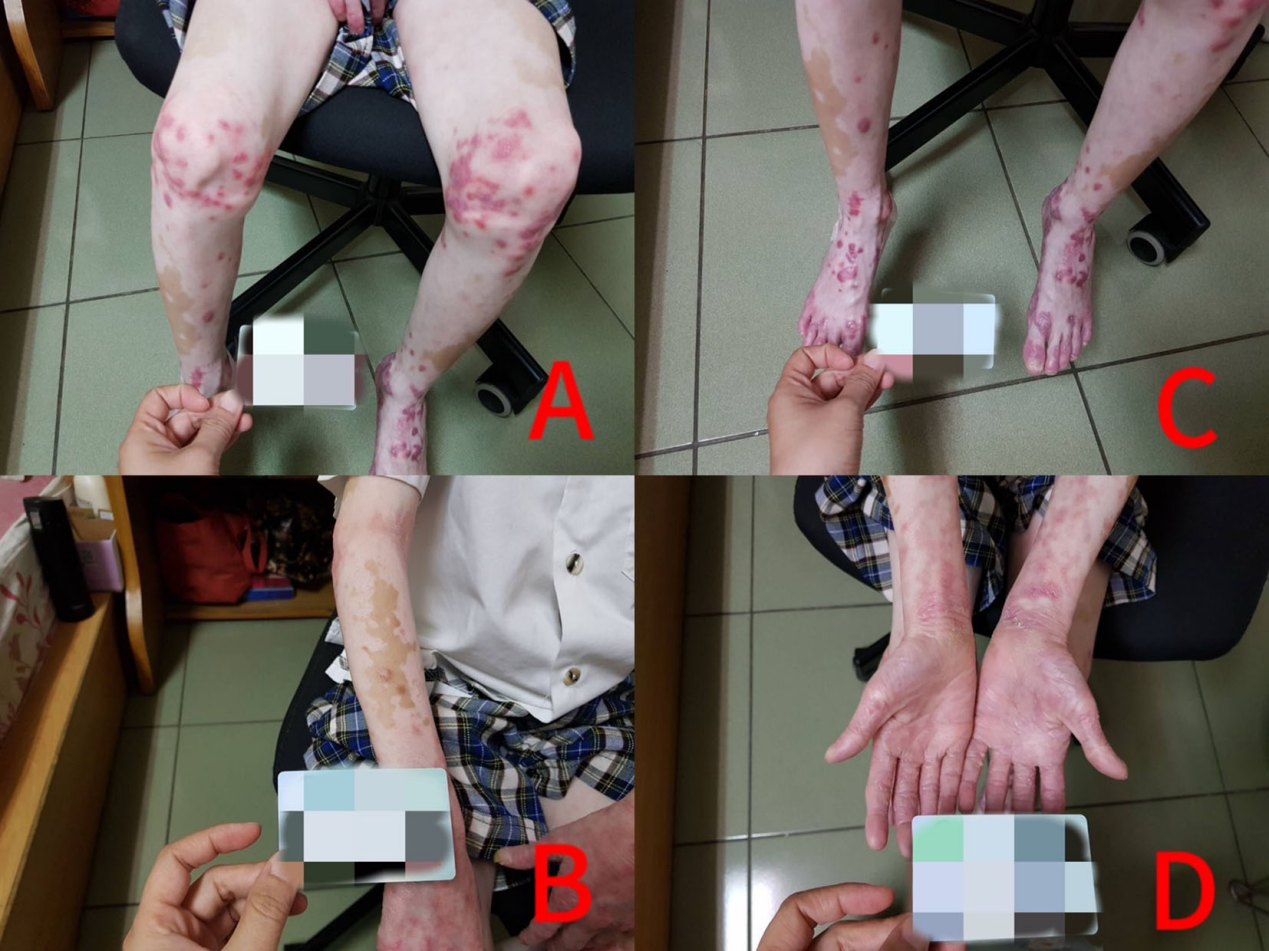
Panels **(A–D)**: Generalized co-occurrence of vitiligo and psoriasis before treatment.

**Figure 2:**
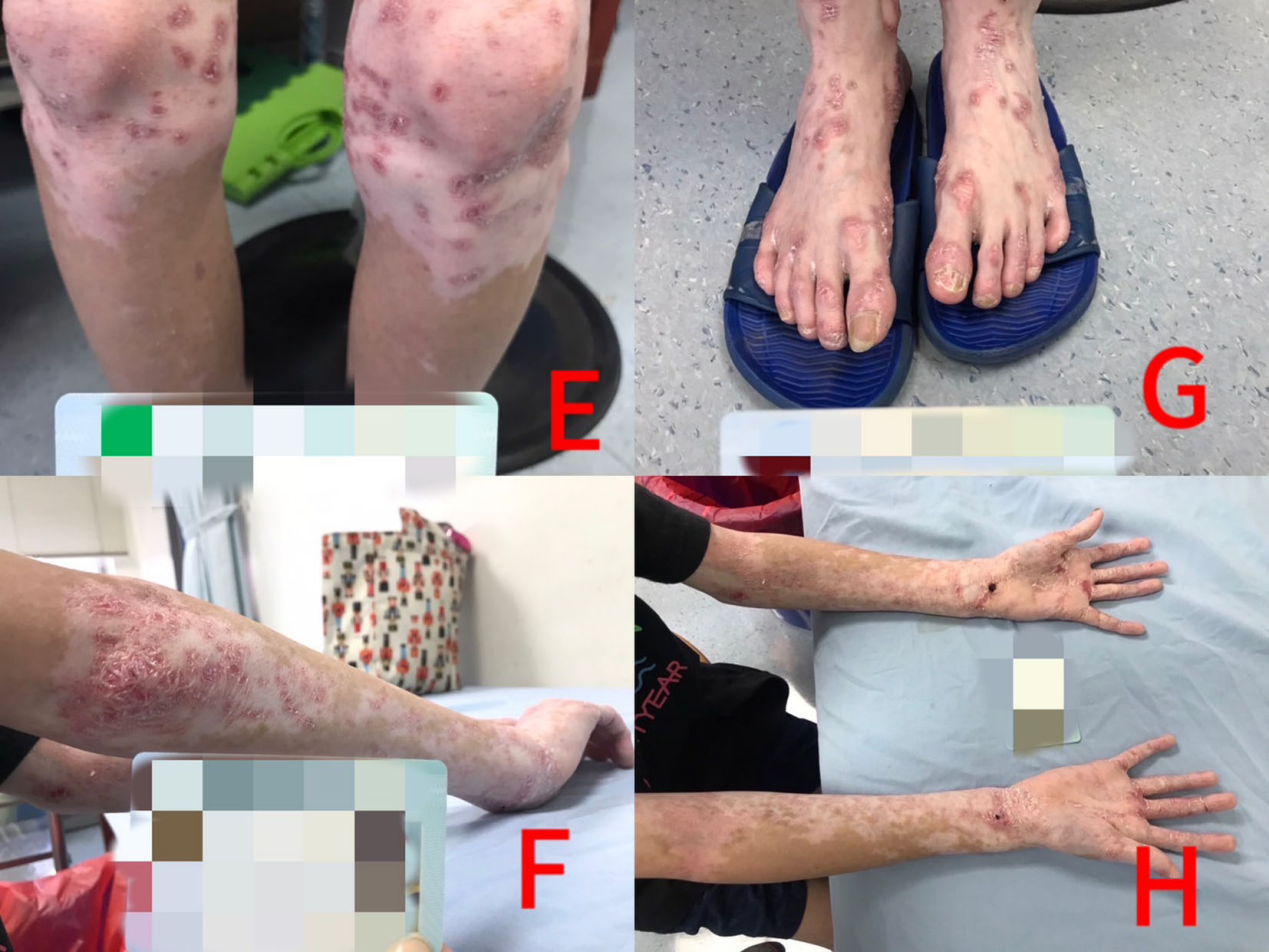
Panels **(E–H)**: Vitiligo responded better than psoriatic lesions to low-dose methotrexate treatment after 3 years.

At 5 years of age, a young boy with no family history of PsO or vitiligo presented with psoriatic lesions on his elbows, knees, hands, and feet, along with associated nail changes. Initially, he was managed with topical corticosteroids at local clinics for his condition. One year later, at 6 years of age, vitiligo appeared to be generalized and included lesions of PsO that extended to the patient’s face and neck. At 7 years of age, he was brought to our outpatient department due to progressed vitiligo and PsO despite the addition of narrow-band ultraviolet B (NB-UVB) phototherapy for 3 months. A physical examination revealed vitiligo lesions all over the patient’s face, neck, hands, trunk, four limbs, palms, and soles, with typical psoriasis plaques on the patient’s knees, elbows, hands, and feet. In regard to psoriatic nails, crumble of more than half the nail bed area was observed on all 10 toenails, and the fingernails were also affected, with mild pitting and minimal onycholysis observed, but no subungual hyperkeratosis or crumble. No musculoskeletal symptoms or signs and no arthritis were observed at that time. All the hematologic, biochemical, and autoimmune examination results were within the normal range. He did not have the budget for treatment with biologics. Hence, our treatment target was focused only on his PsO and psoriatic nails, with low-dosage MTX (5 mg per week owing to his body surface area). During the initial 6-month period, monthly follow-up assessments were conducted to track treatment outcomes and potential side effects. Encouragingly, no adverse effects were observed, and the patient maintained normal levels of blood cells and normal liver function. PsO lesions improved approximately 4 weeks after treatment, and the vitiligo lesion improved approximately 8 weeks after treatment. These positive results underscore the favorable tolerability and effectiveness of the treatment regimen, and the follow-up interval was extended to every 2 months (for 6 months) and finally to every 3 months (to date). After 3 years of treatment at the same dosage of MTX, the psoriatic skin and nail lesions have improved slowly, the psoriasis area severity index (PASI) score has declined from 6.1 to 1.9, and the vitiligo has improved dramatically over time, with the vitiligo area scoring index (VASI) decreasing from 54.8 to 21.5, and with no signs or symptoms of arthritis observed during this period. The psoriatic nail lesions also improved slowly (approximately 18 months after treatment), and improved only partially after 3 years of treatment, with the right big toenail regrowing almost fully, approximately 50% of the nail bed area on the left big toenail regrowing, and the crumbling areas on the other toenails decreasing by approximately 30%, with only minimal residual pitting observed over all fingernails (**Figures 1**, **2**).

## Discussion

PsO is a chronic inflammatory skin disease characterized by well-demarcated erythematous plaques covered by excessive keratinocyte exfoliation. According to the Global Burden of Disease (GBD) Study 2019, its prevalence rate is 57.8 per 100,000 people and it is more common in Northern European nations (1). Vitiligo is an autoimmune disorder that causes the patchy loss of skin pigmentation by attacking melanocytes. It is less prevalent among Asian Americans than among individuals of other races (2). The co-occurrence of PsO and vitiligo was first reported by Selenyi in 1955 (3). PsO has a strong tendency to co-occur with other autoimmune/immune-mediated diseases, and research indicates that PsO patients may have a 25% chance of developing vitiligo during their lifetime (4). Similarly, PsO is the second most common comorbidity in vitiligo patients (5). Therefore, the co-occurrence of PsO and vitiligo is more frequent in individuals in Western countries than in Asian children. Until now, only six cases in which PsO co-occurred with vitiligo have been reported in Asian patients. These comprised four Korean patients, one Japanese patient, and one Chinese patient; the two Korean patients were adults and had previously been reported on in a study written in Korean (6, 7); of the other four patients, who were reported on in a study written in English, two were Korean adults (8, 9). In the Japanese patient, initial symptoms of vitiligo presented in childhood, and PsO co-occurred when she was 66 years old (10). The co-occurrence of PsO and vitiligo under the age of 18 years was observed in only the Chinese patients. The Chinese patient showed initial symptoms of vitiligo at 10 years of age, with PsO co-occurring 2 months later (11). Our patient is the youngest Asian child in whom the initial symptoms of co-occurrence of PsO and vitiligo presented below the age of 6 years.

Current studies have revealed that the interleukin (IL)-23/IL-17 pathogenic axis drives PsO. The activation of plasmacytoid dendritic cells promotes myeloid dendritic cell maturation and the production of tumor necrosis factor-α (TNF-α), IL-12, and IL-23, which leads to the activation of Th (T helper) 1 and Th17, and the subsequent secretion of inflammatory cytokines, such as TNF-α, IL-17, IL-21, and IL-22. These cytokines (especially IL-17) then activate keratinocytes and produce antimicrobial peptides, cytokines, and chemokines, contributing to inflammation amplification (12). Therefore, in addition to conventional synthetic diseases modified antirheumatic drugs (csDMARD), biologics are commonly used in treating PsO, including TNF inhibitor (TNFi), anti-IL-17 antibodies, anti-IL-17 receptor antibodies, anti-IL12/23 antibodies, anti-IL-23 antibodies, and Janus kinase inhibitor (JAKi).

Before 2013, no positive results associated with treating vitiligo using MTX had been published (13); only after 2015 did a few studies with a small number of patients reveal the effect of MTX in vitiligo (14, 15), and since 2020 a few positive results associated with MTX combined with low-dose steroids or phototherapy in vitiligo have also been reported in a small number of cases (16, 17). Yen et al. previously reported that PsO and vitiligo have similar pathogenic backgrounds. They are both cell-mediated Th1 diseases in which elevated TNF-α and interferon (INF) levels and the activation of the Th17 pathway are observed (18). After that, TNFi, anti-IL-17 antibodies, anti-IL-23 antibodies, and JAKi are reasonable in treating the co-occurrence of PsO and vitiligo. In recent vitiligo studies, the IFN-γ- CXCL9/10-CXCR3 axis appears important in vitiligo via inhibiting melanogenesis, inducing apoptosis of melanocytes, and further recruiting T cells to the skin. These are all involved in the JAK/signal transducer and activator of the transcription (STAT) pathway. In addition, cytokines, including heat shock protein 70i (HSP70i), IL-15, IL-17/23, and TNF, and also the wnt signaling pathway, regulatory T cells (Tregs), and micro-RNA (miRNAs), have also been proven to be involved in the pathogenesis of vitiligo. Based on the pathophysiology of vitiligo, the current treatment for moderate to severe vitiligo includes TNFi, anti-IL-17 antibody, anti-IL-23 antibody, and JAKi (19). However, many case reports have revealed that the use of these biologics in treating PsO or the co-occurrence of PsO and vitiligo, except for JAKi combined with phototherapy (20, 21), might induce the new onset of or worsen pre-existing vitiligo (22, 23). The first case of vitiligo successfully treated with JAKi was reported in 2015 (24). Later, positive results of topical JAKi in vitiligo were published in 2017 (25). Nevertheless, it was not until July 2022 that the Food and Drug Administration (FDA) approved topical ruxolitinib as a treatment for non-segmental vitiligo in patients above the age of 12 years. Although some oral JAKi studies in vitiligo had positive results, no oral JAKi are currently approved for treatment. In addition, the FDA-approved label for topical ruxolitinib features a pointed reminder that the relationship between the JAKs and their inhibition and vitiligo is not entirely understood. On the label it is stated that the “relevance of inhibition of specific JAK enzymes to therapeutic effectiveness is unknown.”

The efficacy of MTX in treating rheumatoid arthritis, psoriatic arthritis, and PsO was previously thought to be inherent to its action as a competitive inhibitor of dihydrofolate reductase (DHFR); enzymatic inhibition reduces the intracellular levels of downstream folate pathway intermediates required for nucleotide synthesis, and this results in impaired DNA replication. A possible new mechanism of MTX in immune disease, namely its inhibition of the JAK/STAT pathway, was proposed in 2015 (26). MTX has multiple mechanisms that potentially contribute to anti-inflammatory actions, including (a) inhibition of purine and pyrimidine synthesis, (b) transmethylation reactions, (c) the translocation of nuclear factor-κB (NF-κB) to the nucleus, (d) signaling via the JAK/STAT pathway, (e) nitric oxide production, and (f) the promotion of adenosine release and expression of specific long non-coding RNAs. Among these possible mechanisms, (c), (d), and (e) have been proposed to be the principal anti-inflammatory mechanisms (27). The (f) mechanism of anti-inflammation was previously included in the main mechanism, but a recent study revealed that the polymorphism of the genes involved in adenosine production and signaling could influence the response to MTX (28). The patient reported on in our case report started taking MTX in 2018; at that time, MTX was the first choice of first-line csDMARDs for moderate to severe PsO and was not considered helpful for vitiligo treatment, and JAKi, in both topical and oral form, had still not been approved for the treatment of vitiligo, especially in children under the age of 12 years. Therefore, in addition to presenting the youngest patient with co-occurrence of vitiligo and PsO, in our case report we present the successful treatment with MTX, especially of vitiligo and not just of PsO, as a piece of minor evidence that the JAK/STAT pathway may play an important role in both vitiligo and PsO.

Another interesting observation is that in our patient the PsO lesions were all found within the later onset vitiligo lesion area. From Sharquie’s report, 15 patients were found to have co-occurrence of PsO and vitiligo. The PsO lesions of eight patients were in the vitiligo lesions, and in another nine their PsO lesions were separate from the vitiligo lesions (29). Whether or not the excellent response of PsO and vitiligo to MTX in our patient is related to the fact that these two skin lesions (for two conditions with a common pathologic pathway) were in the same area still needs further study.

## Conclusion

Li et al. summarized three possible mechanisms of the co-occurrence of vitiligo and PsO: (a) isomorphic reaction, (b) shared genetic basis of autoimmunity and inflammation, and (c) shared cellular immune pathways and related cellular molecules (11). Based on the occurrence of vitiligo following the onset of PsO, the lack of a traceable family history of PsO or vitiligo, and the good results with MTX treatment in both PsO and vitiligo in our case, we suggest that the co-occurrence of PsO and vitiligo is not a coincidence and may be related to the shared cellular immune pathways of both diseases. Although the effects of TNFi and anti-IL-17 antibodies on vitiligo are variable, the utilization of MTX in conjunction with steroids or phototherapy and the consideration of JAKi targeting the JAK/STAT pathway hold potential for treating vitiligo. However, it is important to note that the hypothesis regarding the involvement of the JAK/STAT pathway in vitiligo treatment needs to be substantiated with further evidence.

## Data availability statement

The original contributions presented in the study are included in the article/supplementary material. Further inquiries can be directed to the corresponding author.

## Ethics statement

Written informed consent was obtained from the individual(s) for the publication of any potentially identifiable images or data included in this article.

## Author contributions

A-PH: Resources, Supervision, Writing – review & editing, Validation. P-YL: Writing – original draft, Investigation. T-MC: Writing – original draft, Investigation. JW: Supervision, Validation, Writing – review & editing.

## References

[1] Damiani G Bragazzi NL Aksut CK Wu D Alicandro G McGonagle D. The global, regional, and national burden of psoriasis: Results and insights from the global burden of disease 2019 study. Front Med (Lausanne) (2021) 16:743180. doi: 10.3389/fmed.2021.743180 PMC8716585 34977058

[2] Mastacouris N Strunk A Garg A. Incidence and prevalence of diagnosed vitiligo according to race and ethnicity, age, and sex in the US. JAMA Dermatol (2023) 19:e232162. doi: 10.1001/jamadermatol.2023.2162 PMC10357354 37466934

[3] Selenyi A. Vitiligo and psoriasis on the same side with syringomyelia. Borgyogy Venerol Sz. (1955) 9:94–6. 13250048

[4] Masood S Sajid S Jafferani A Tabassum S Ansar S. Multiple autoimmune syndromes associated with psoriasis; a. rare clinical presentation. Oman Med J (2014) 29(2):130–1. doi: 10.5001/omj.2014.31 PMC3976724 24715941

[5] Sheth VM Guo Y Qureshi AA. Comorbidities associated with vitiligo: A ten-year retrospective study. Dermatology. (2013) 227(4):311–5. doi: 10.1159/000354607 24107643

[6] Lee HC Lim SW Suh MK Choi JH Kwon SW Lee JW. A case of psoriasis vulgaris associated with vitiligo. Korean J Dermatol (2003) 41:1416–8.

[7] Kim YJ Kang HY Lee ES Kim YC. A case of psoriasis strictly localized on a vitiligo lesion. Korean J Dermatol (2006) 44:528–30.

[8] Park JM Kim HJ Bae BG Park YK. A case of concurrent vitiligo and psoriasis. Ann Dermatol (2009) 21:330–3. 10.5021/ad.2009.21.3.330 PMC2861223 20523818

[9] Lee SH Kim YS Kim HJ Park YL. Psoriasis, vitiligo and crohn’s disease co-existing in a single patient: A variant type of multiple autoimmune syndrome? Ann Dermatol (2017) 29(6):782–5. doi: 10.5021/ad.2017.29.6.782 PMC5705363 29200770

[10] Ono S Tanizaki H Otsuka A Endo Y Koyanagi I Kataoka TR. Coexistent skin lesions of vitiligo and psoriasis vulgaris. Immunohistochemical analyses for IL-17A-producing cells and regulatory T cells. Acta Derm Venereol. (2014) 94(3):329–30. doi: 10.2340/00015555-1713 24129673

[11] Li R Zhang JA Jiang YQ Wu MZ Chen K. Co-occurrence of vitiligo and psoriasis in an 11-year-old girl: a case report. Int J Dermatol Venereo. (2020) 3:4. doi: 10.1097/JD9.0000000000000047

[12] Zhou X Chen Y Cui L Shi Y Guo C. Advances in the pathogenesis of psoriasis: from keratinocyte perspective. Cell Death Dis (2022) 13:81. doi: 10.1038/s41419-022-04523-3 35075118 PMC8786887

[13] Alghamdi K Khurrum H. Methotrexate for the treatment of generalized vitiligo. Saudi Pharm J (2013) 21:423–4. doi: 10.1016/j.jsps.2012.12.003 PMC3824946 24227963

[14] Singh H Kumaran MS Bains A Parsad D. A randomized comparative study of oral corticosteroid minipulse and low-dose oral methotrexate in the treatment of unstable vitiligo. Dermatology. (2015) 231:286–90. doi: 10.1159/000433424 26278124

[15] Garza-Mayers AC Kroshinsky D. Low-dose methotrexate for vitiligo. J Drugs Dermatol (2017) 16:705–6. 28697225

[16] ElGhareeb MI Metwalli M AbdelMoneim N. Combination of oral methotrexate and oral mini-pulse dexamethasone vs either agent alone in vitiligo treatment with follow up by dermoscope. Dermatol Ther (2020) 33:e13586. doi: 10.1111/dth.13586 32410362

[17] Tavitova A Valle Y Lomonosov K. Using methotrexate in the treatment of advanced Vitiligo. J Cosmet Dermatol (2023) 22:1136–8. doi: 10.1111/jocd.15524 36409563

[18] Yen H Chi CC. Association between psoriasis and vitiligo: A systematic review and. meta-analysis. Am J Clin Dermatol (2019) 20(1):31–40. doi: 10.1007/s40257-018-0394-1 30317450

[19] Feng Y Lu Y. Advances in vitiligo: Update on therapeutic targets. Front Immunol (2022) 13:986918. doi: 10.3389/fimmu.2022.986918 36119071 PMC9471423

[20] Liu LY Strassner JP Refat MA Harris JE King BA. Repigmentation in vitiligo using the Janus kinase inhibitor tofacitinib may require concomitant light exposure. J Am Acad Dermatol (2017) 77(4):675–82. doi: 10.1016/j.jaad.2017.05.043 PMC6233876 28823882

[21] Tajalli M Kabir S Vance TM Qureshi AA. Effective use of oral tofacitinib and phototherapy in a patient with concomitant alopecia areata, vitiligo, and plaque and inverse psoriasis. Clin Case Rep (2020) 8:819–22. 10.1002/ccr3.2759 PMC7250974 32477525

[22] Burlando M Muracchioli A Cozzani E and Parodi A. Psoriasis, vitiligo, and biologic therapy: Case report and narrative review. Case Rep Dermatol (2021) 13:372–8. doi: 10.1159/000514198 PMC8339517 34413735

[23] Su HJ Chan YP Shen PC Ku CL Ng CY. Anti-IL-17A antibody-associated *de novo.* vitiligo: Case report and review of literature. Front Immunol (2023) 13:1077681. doi: 10.3389/fimmu.2022.1077681 36741377 PMC9889818

[24] Craiglow BG King BA. Tofacitinib citrate for the treatment of vitiligo: A pathogenesis-directed therapy. JAMA Dermatol (2015) 151:1110–2. doi: 10.1001/jamadermatol.2015.1520 26107994

[25] Rothstein B Joshipura D Saraiya A Abdat R Ashkar H Turkowski Y. Treatment of vitiligo with the topical Janus kinase inhibitor ruxolitinib. J Am Acad Dermatol (2017) 76:1054–60.e1. doi: 10.1016/j.jaad.2017.02.049 28390737

[26] Thomas S Fisher KH Snowden JA Danson SJ Brown S Zeidler MP. Methotrexate is a JAK/STAT pathway inhibitor. PLoS One (2015) 10:e0130078. doi: 10.1371/journal.pone.0130078 26131691 PMC4489434

[27] Cronstein BN Aune TM. Methotrexate and its mechanisms of action in inflammatory arthritis. Nat Rev Rheumatol (2020) 16:145–54. doi: 10.1038/s41584-020-0373-9 32066940

[28] Singh A Gangadharan H Gupta V Patro PS Misra R Aggarwal A. Polymorphism of genes involved in methotrexate pathway: Predictors of response to methotrexate therapy in Indian rheumatoid arthritis patients. Int J Rheum Dis (2021) 24:654–62. doi: 10.1111/1756-185X.14100 33780152

[29] Sharquie KE Salman HA Yaseen AK. Psoriasis and vitiligo are close relatives. Clin Cosmet Investig Dermatol (2017) 10:341–5. doi: 10.2147/CCID.S142819 PMC5587085 28919796

